# Supplementary material for: Alterations in Alzheimer’s Disease-Associated Gene Expression in Severe Obstructive Sleep Apnea Patients
Source: J Clin Med. 2019 Sep 1;8(9):1361. doi: 10.3390/jcm8091361 (PMC6780075; doi:10.3390/jcm8091361)
Supplement: Supplementary file 1 [file jcm-08-01361-s001.pdf]

Supplementary Online Content

Table S1. Genes and pseudogenes associated with Alzheimer's disease.

Gene Symbol	Name	Gene Length	Onset of Alzheimer's Disease	ENSEMBL Gene ID
APP	amyloid beta (A4) precursor protein	2326.72	early	ENSG00000142192
FKBP1B	FK506 binding protein 1B	929.42	early	ENSG00000119782
PSEN1	presenilin 1	3941.76	early	ENSG00000080815
PSEN2	presenilin 2	1389.34	early	ENSG00000143801
ABCA1	ATP-binding cassette, sub-family A (ABC1), member 1	10494	late	ENSG00000165029
ABCA7	ATP-binding cassette, sub-family A (ABC1), member 7	1468.3	late	ENSG00000064687
ANK1	ankyrin 1, erythrocytic	2108.51	late	ENSG00000029534
APOC1	apolipoprotein C-I	446.35	late	ENSG00000130208
APOE	apolipoprotein E	1124.92	late	ENSG00000130203
BACE2	beta-site APP-cleaving enzyme 2	2372.29	late	ENSG00000182240
BCR	breakpoint cluster region	2509.52	late	ENSG00000186716
BIN1	bridging integrator 1	1549.99	late	ENSG00000136717
CASS4	Cas scaffolding protein family member 4	2883.85	late	ENSG00000087589
CCL2	chemokine (C-C motif) ligand 2	753.22	late	ENSG00000108691
CD2AP	CD2-associated protein	5412	late	ENSG00000198087
CD33	CD33 molecule	1613.85	late	ENSG00000105383
CELF1	CUGBP, Elav-like family member 1	4778.25	late	ENSG00000149187
CLU	clusterin	607.12	late	ENSG00000120885
COX4I1	cytochrome c oxidase subunit IV isoform 1	711.38	late	ENSG00000131143

CR1	complement component (3b/4b) receptor 1 (Knops blood group)	7442.25	late	ENSG00000203710
CXCL8	chemokine (C-X-C motif) ligand 8	1248.62	late	ENSG00000169429
DSG2	desmoglein 2	5831	late	ENSG00000046604
EPHA1	EPH receptor A1	2181.3	late	ENSG00000146904
EPHA10	EPH receptor A10	3964	late	ENSG00000183317
FAM136A	family with sequence similarity 136, member A	1794.23	late	ENSG00000035141
FERMT2	fermitin family member 2	2678.61	late	ENSG00000073712
GAS7	growth arrest-specific 7	4414.8	late	ENSG00000007237
GRIK2	glutamate receptor, ionotropic, kainate 2	4185	late	ENSG00000164418
GRN	granulin	1084.03	late	ENSG00000030582
HAS3	hyaluronan synthase 3	1342.72	late	ENSG00000103044
HLA-A	major histocompatibility complex, class I, A	5422	late	ENSG00000206503
HLA-DRB1	major histocompatibility complex, class II, DR beta 1	1229	late	ENSG00000196126
HLA-DRB5	major histocompatibility complex, class II, DR beta	1260	late	ENSG00000198502
IL1RN	interleukin 1 receptor antagonist	1776.01	late	ENSG00000136689
IL6	interleukin 6	885.33	late	ENSG00000136244
ITGAL	integrin, alpha L (antigen CD11A (p180), lymphocyte function-associated antigen 1; alpha polypeptide)	1875.14	late	ENSG00000005844
KLK2	kallikrein-related peptidase 2	528	late	ENSG00000167751
LRP2	low density lipoprotein receptor-related protein 2	3156	late	ENSG00000081479

MAPT	microtubule-associated protein tau	2987.74	late	ENSG00000186868
MEF2C	myocyte enhancer factor 2C	3003	late	ENSG00000081189
MS4A4	membrane-spanning 4-domains, subfamily A, member 8	1353	late	ENSG00000166959
MS4A6E	membrane-spanning 4-domains, subfamily A, member 6E	711	late	ENSG00000166926
PICALM	phosphatidylinositol binding clathrin assembly protein	1679.44	late	ENSG00000073921
PIN1	peptidylprolyl cis/trans isomerase, NIMA-interacting 1	1193.02	late	ENSG00000127445
PILRA	paired immunoglobin-like type 2 receptor alpha	1302.4	late	ENSG00000085514
PLD3	phospholipase D family, member 3	1163.79	late	ENSG00000105223
PRSS42	protease, serine, 42	1280	late	ENSG00000178055
PRSS45	protease, serine, 45	3128	late	ENSG00000188086
PTK2B	protein tyrosine kinase 2 beta	3722.59	late	ENSG00000120899
RIN3	Ras and Rab interactor 3	3419.45	late	ENSG00000100599
SEC31A	SEC31 homolog A, COPII coat complex component	2083.44	late	ENSG00000138674
SLC22A2	solute carrier family 22 (organic cation transporter), member 2	2597	late	ENSG00000112499
SLC24A4	solute carrier family 24 (sodium/potassium/calcium exchanger), member 4	9950	late	ENSG00000140090
SORL1	sortilin-related receptor, L (DLR class) A repeats containing	10009.75	late	ENSG00000137642

STOX2	Storkhead Box 2	3626.57	late	ENSG00000173320
THNSL2	threonine synthase-like 2	1609.65	late	ENSG00000144115
TMIE	transmembrane inner ear	1824	late	ENSG00000181585
TRDMT1	TRNA aspartic acid methyltransferase 1	3705.83	late	ENSG00000107614
TREM2	triggering receptor expressed on myeloid cells 2	1044	late	ENSG00000095970
ZCWPW1	zinc finger, CW type with PWWP domain 1	1893.78	late	ENSG00000078487

Table S2. Summary of the expressions of 60 Alzheimer's disease-associated genes and pseudogenes in the severe OSA and non-severe OSA groups in descending order by probability.

Gene Symbol	Expression		Log_2(Fold Change)	Regulation	Probability
	Severe OSA	Non-Severe OSA			
CCL2	12.992	116.716	-3.167	Down	0.868[a]
IL6	2.854	33.06	-3.534	Down	0.857[a]
CXCL8	2.042	20.058	-3.296	Down	0.824[a]
HLA-A	1.272	14.006	-3.460	Down	0.811[a]
IL1RN	239.35	57.236	2.064	Up	0.806[a]
HLA-DRB1	41.398	14.398	1.523	Up	0.716
ITGAL	6.81	1.944	1.808	Up	0.635
HLA-DRB5	22.194	10.358	1.099	Up	0.617
CLU	490.412	308.192	0.670	Up	0.522
MS4A4	2.176	4.908	-1.173	Down	0.512
APP	382.418	587.644	-0.619	Down	0.503
TRDMT1	2.702	5.57	-1.043	Down	0.502
ANK1	3.87	1.878	1.043	Up	0.464
CR1	1.076	0.214	2.329	Up	0.439
EPHA1	6.552	4.254	0.623	Up	0.408
MS4A6E	0.39	1.174	-1.590	Down	0.399
BACE2	69.39	90.6	-0.385	Down	0.390
ABCA7	3.636	2.218	0.713	Up	0.385
MAPT	1.134	0.408	1.475	Up	0.382
PTK2B	9.514	6.938	0.456	Up	0.376
DSG2	24.424	31.632	-0.373	Down	0.375
GAS7	5.698	3.952	0.528	Up	0.370
PILRA	1.406	2.452	-0.802	Down	0.370
FERMT2	14.036	17.708	-0.335	Down	0.348
BIN1	23.218	18.884	0.298	Up	0.335
CASS4	13.794	16.864	-0.290	Down	0.323
APOE	24.526	29.308	-0.257	Down	0.318
SEC31A	136.526	159.614	-0.225	Down	0.311
CD33	1.416	2.06	-0.541	Down	0.304
PLD3	121.136	139.682	-0.206	Down	0.300

PIN1	23.774	27.412	-0.205	Down	0.291
GRN	133.2	116.384	0.195	Up	0.289
PSEN1	12.776	14.876	-0.220	Down	0.286
RIN3	2.84	2.234	0.346	Up	0.276
SORL1	8.136	9.338	-0.199	Down	0.267
PSEN2	7.204	6.208	0.215	Up	0.265
APOC1	6.362	7.216	-0.182	Down	0.252
ABCA1	1.152	0.866	0.412	Up	0.248
COX4I1	142.382	153.196	-0.106	Down	0.240
CELF1	9.536	8.628	0.144	Up	0.240
PICALM	56.394	52.512	0.103	Up	0.233
TREM2	0.856	1.076	-0.330	Down	0.232
FAM136A	15.05	16.288	-0.114	Down	0.232
BCR	21.86	20.414	0.099	Up	0.228
SLC22A2	0.056	0.01	2.485	Up	0.224
ZCWPW1	1.442	1.674	-0.215	Down	0.223
MEF2C	16.674	15.664	0.090	Up	0.221
CD2AP	19.416	20.49	-0.078	Down	0.217
FKBP1B	3.786	4.126	-0.124	Down	0.216
HAS3	5.378	5.772	-0.102	Down	0.214
EPHA10	0.476	0.576	-0.275	Down	0.207
SLC24A4	0.202	0.274	-0.440	Down	0.205
THNSL2	8.034	7.658	0.069	Up	0.204
LRP2	0.288	0.22	0.389	Up	0.203
PRSS45	0.054	0.022	1.295	Up	0.196
GRIK2	0.312	0.368	-0.238	Down	0.196
TMIE	0.112	0.084	0.415	Up	0.190
KLK2	0.448	0.49	-0.129	Down	0.186
STOX2	2.428	2.48	-0.031	Down	0.180
PRSS42	0.036	0.022	0.710	Up	0.180

Abbreviations: OSA, obstructive sleep apnea. [a]Significantly up- and down-regulated genes were defined as FDR ≤0.001 (i.e. probability ≥0.8) and an absolute value of Log_2Ratio ≥1.

Table S3. Results of Gene Ontology related to five Alzheimer's disease-associated differentially expressed genes in severe OSA patients.

Term	Count	Percentage	*p*-Value	Adjusted *p*-value
Biological process				
Immune response	5	1.3	<0.001	<0.001[a]
Cellular response to interleukin-1	3	0.8	<0.001	0.01[a]
Cellular response to tumor necrosis factor	3	0.8	<0.001	0.02[a]
Cellular response to lipopolysaccharide	3	0.8	<0.001	0.01[a]
Regulation of vascular endothelial growth factor production	2	0.5	0.001	0.04[a]
Negative regulation of interleukin-1-mediated signaling pathway	2	0.5	0.001	0.04[a]
PERK-mediated unfolded protein response	2	0.5	0.003	0.10
Inflammatory response	3	0.8	0.003	0.09
Cellular response to dexamethasone stimulus	2	0.5	0.007	0.17
Cellular response to fibroblast growth factor stimulus	2	0.5	0.007	0.16
Response to amino acid	2	0.5	0.007	0.15
Response to antibiotic	2	0.5	0.008	0.14
Acute-phase response	2	0.5	0.009	0.15
Monocyte chemotaxis	2	0.5	0.01	0.15
Response to heat	2	0.5	0.01	0.16
Humoral immune response	2	0.5	0.01	0.18
Response to glucocorticoid	2	0.5	0.02	0.19
Neutrophil chemotaxis	2	0.5	0.02	0.18
Chemokine-mediated signaling pathway	2	0.5	0.02	0.18
Defense response to Gram-positive bacterium	2	0.5	0.02	0.21
Chemotaxis	2	0.5	0.03	0.27
Cytokine-mediated signaling pathway	2	0.5	0.03	0.28
Regulation of cell shape	2	0.5	0.03	0.28
Aging	2	0.5	0.04	0.31

Positive regulation of ERK1 and ERK2 cascade	2	0.5	0.04	0.32
Angiogenesis	2	0.5	0.05	0.37
Negative regulation of cell proliferation	2	0.5	0.09	0.55
Cellular component				
Extracellular space	4	1.0	0.002	0.046[a]
Intracellular	3	0.8	0.03	0.37
Extracellular region	3	0.8	0.04	0.35
Molecular function				
Chemokine activity	2	0.5	0.01	0.22
Cytokine activity	2	0.5	0.04	0.36
Receptor binding	2	0.5	0.08	0.45

Abbreviations: OSA, obstructive sleep apnea; [a]*P*-values <0.05 adjusted using the Benjamini method were statistically significant.
